# The molecular logic of early metastasis in pancreatic cancer: crosstalk between tumor and microenvironment

**DOI:** 10.3389/fcell.2025.1726581

**Published:** 2025-11-28

**Authors:** Andrea Costamagna, Noemi Ghiglione, Miriam Martini

**Affiliations:** Department of Molecular Biotechnology and Health Sciences, University of Torino, Turin, Italy

**Keywords:** pancreatic ductal adenocarcinoma, early metastasis, tumor microenvironment, epithelial–mesenchymal plasticity, pre-metastatic niche, PDAC

## Abstract

Pancreatic ductal adenocarcinoma (PDAC) exhibits early systemic dissemination that precedes clinical detection, challenging the classical model of metastasis as a late evolutionary event. Mounting evidence indicates that molecular and cellular programs enabling invasion and distant colonization emerge at preinvasive or early carcinoma stages. This mini-review synthesizes recent advances defining how tumor-intrinsic mechanisms, such as KRAS-driven basal extrusion, epithelial–mesenchymal plasticity and chromosomal instability, cooperate with microenvironmental cues to promote early metastatic competence. The desmoplastic stroma, rich in fibroblasts, inflammatory mediators and aligned extracellular matrix, provides both structural and biochemical support for tumor-cell escape. Additionally, neural and immune interactions, including chemokine and cytokine signaling, facilitate perineural invasion and systemic pre-metastatic niche (PMN) conditioning via extracellular vesicles and cytokine networks. Recognizing PDAC as a systemic disease from inception reframes therapeutic priorities: early systemic therapy and biomarker-guided patient stratification may intercept occult dissemination. We propose that integrating mechanistic insights on tumor–microenvironment crosstalk with perioperative and liquid-biopsy-driven clinical strategies could redefine early intervention in PDAC.

## Introduction

Pancreatic ductal adenocarcinoma (PDAC) is a biologically recalcitrant malignancy with a 5-year survival of approximately 12%. Approximately half of patients present with metastatic disease at the time of diagnosis (Siegel et al., 2024; Halbrook et al., 2023). Despite advances in diagnostic imaging, surgical techniques and systemic therapies, the majority of cases are detected at advanced stages. Even when resection is technically feasible, early recurrence and systemic dissemination are common, consistent with the view that PDAC undergoes early dissemination at the molecular level, preceding clinical detection (Sohal et al., 2014).

Traditionally, metastasis has been framed as a late event in tumor evolution, occurring after accumulation of sufficient genetic and epigenetic alterations (Yachida et al., 2010). However, accumulating evidence in PDAC challenges this linear model. Recent studies have revealed substantial interpatient heterogeneity in evolutionary trajectories with no single dominant metastatic pattern (Notta et al., 2016; Pei et al., 2025), supporting the concept that dissemination can occur at early, potentially preinvasive, stages in a process less influenced by inherent genomic features (Chan-Seng-Yue et al., 2020) and more by crosstalk between cancer cells and the local tumor microenvironment (TME).

These processes involve not only canonical oncogenic drivers, such as *KRAS*, but also dynamic interactions with stromal, immune and vascular compartments that collectively facilitate tumor-cell dissemination and colonization of distant sites. In parallel, pancreatic cancer cells exhibit marked phenotypic plasticity, enabling adaptation to changing environmental cues and exploitation of systemic signals to prime pre-metastatic niches (PMN) (Pei et al., 2025; Ren et al., 2018; Ma et al., 2022; Crawford et al., 2019). The early establishment of a pro-metastatic microenvironment often remains clinically silent until overt metastases manifest. Elucidating how tumor-intrinsic programs and extrinsic microenvironmental cues cooperate to drive early systemic spread is critical for developing biomarkers and interventions that intercept PDAC at its most vulnerable stages.

In this mini-review, we synthesize emerging evidence on the molecular interactions that govern early metastasis in PDAC, with emphasis on the interplay between tumor signaling pathways and microenvironmental factors. We outline how this crosstalk shapes the metastatic trajectory and discuss implications for future therapeutic strategies. The following sections examine mechanistic drivers and microenvironmental programs that coordinate early dissemination and metastatic colonization in PDAC.

## The problem at a glance

Metastasis initiates when a subset of cells within the primary lesion acquires the capacity to invade surrounding tissues and disseminate. This behavior is markedly heterogeneous both within individual tumors and across patients (Vogelstein et al., 2013; Gerstberger et al., 2023). In PDAC, early genomic reconstructions of matched primaries and metastases supported a predominantly late-dissemination model (Yachida et al., 2010).

This view was challenged by mathematical modeling suggesting that many patients already carry tissue-resident disseminated tumor cells (DTCs) or micrometastases at diagnosis (Haeno et al., 2012). Lineage-tracing studies then showed that epithelial cells can exit preinvasive pancreatic intraepithelial neoplasia (PanIN) lesions and enter the stroma, bloodstream and liver before overt carcinoma appears (Rhim et al., 2012). These early-disseminating cells exhibited features consistent with partial epithelial–mesenchymal transition (EMT), although their capacity for durable outgrowth at distant sites at that stage remained uncertain. Throughout, we use DTCs to denote tissue-resident disseminated cells and circulating tumor cells (CTCs) to denote blood-borne cells.

The emerging early-dissemination paradigm provides a mechanistic context for otherwise puzzling clinical observations, including an estimated 30%–40% rate of metastatic recurrence after resection of apparently small (0.5–2 cm) primaries (Ansari et al., 2017; Agarwal et al., 2008) and occasional detection of occult PDAC at pancreatectomy performed for presumed nonmalignant chronic pancreatitis, with metastasis already present in a subset of patients (Sakorafas and Sarr, 2003). Collectively, the data suggest that a subset of precursor or early carcinoma cells acquires dissemination-competent phenotypes through clonal diversity and context-dependent programs that modulate adhesion, motility and microenvironmental remodeling, enabling breach of local barriers and systemic seeding. We synthesize emerging evidence on the molecular interactions that govern early metastasis in PDAC, with emphasis on the interplay between tumor signaling pathways and microenvironmental factors (Table 1).

**TABLE 1:** Key drivers of early dissemination in PDAC.

Category	Representative Molecules / Pathways	Mechanistic Role	Key References
Genetic/Intrinsic	KRAS, TP53, SMAD4, ARID1A, ATM	Oncogenic signaling, chromosomal instability, subtype plasticity	Makohon-Moore et al. (2017); Chan-Seng-Yue et al. (2020)
Mechanical/Polarity	S1P/S1PR2, p120-catenin	Basal extrusion of viable cells	Hendley et al. (2016)
EMT/Plasticity	Partial EMT, hybrid classical–basal states	Collective invasion, adaptability	Aiello et al. (2018); Chan-Seng-Yue et al. (2020)
ECM Remodeling	FAK, collagen alignment, MMP2/9	Stromal reorganization enabling invasion	Ray et al. (2022); Provenzano et al. (2012)
Fibroinflammatory signaling	IL-6/STAT3, TGF-β/SMAD, CXCL12/CXCR4	Stromal activation, immunosuppression, directed invasion	Ren et al. (2018); Wu et al. (2017)
Perineural invasion	CX3CL1/CX3CR1, SEMA3D, L1CAM	Neural tropism, early spread	Marchesi et al. (2008); Jurcak et al. (2019)
Pre-metastatic niche	Exosomal MIF, IL-6–SAA axis	Liver conditioning, fibronectin deposition	Costa-Silva et al. (2015); Lee et al. (2019)
Dormancy / Immune Editing	ER stress, MHC-I loss	Quiescence and immune evasion of DTCs	Pommier et al. (2018)
Biomarker / Clinical translation	CTCs, ctDNA, EVs	Early detection of occult metastasis	Franses et al. (2020); Maulat et al. (2024)

Summary of major tumor-intrinsic and microenvironmental mechanisms promoting early metastatic spread in pancreatic ductal adenocarcinoma. The table highlights genetic and polarity cues, epithelial-mesenchimal transition (EMT)-related plasticity, stromal and neural interactions, and systemic factors shaping the pre-metastatic niche (PMN) and dormancy, with representative pathways and references.

Summary of major tumor-intrinsic and microenvironmental mechanisms promoting early metastatic spread in pancreatic ductal adenocarcinoma. The table highlights genetic and polarity cues, epithelial-mesenchimal transition (EMT)-related plasticity, stromal and neural interactions, and systemic factors shaping the pre-metastatic niche (PMN) and dormancy, with representative pathways and references.

## Tumor-intrinsic programs driving early dissemination

*Genetic alterations and chromosomal instability.* Early models posited that metastatic competence requires stepwise acquisition of somatic mutations beyond those present in the primary tumor. However, comparative sequencing of matched primaries and metastases shows near-identity for canonical driver alterations (e.g., *KRAS*, *TP53*, *SMAD4*, *ARID1A*, *ATM*), with much of the divergence attributable to presumed passenger events (Makohon-Moore et al., 2017; Connor et al., 2019). Many alterations in the primary tumor are subclonal (cancer cell fraction <1), generating pronounced intratumoral heterogeneity (Yachida et al., 2010; Dentro et al., 2021; Lin et al., 2020). Chromosomal instability (CIN)—manifesting as broad copy-number alterations and structural rearrangements—is pervasive in PDAC and contributes to malignant transformation while creating selectable diversity that can underwrite metastatic behavior (Notta et al., 2016; Chan-Seng-Yue et al., 2020; Maddipati et al., 2022; Bakhoum et al., 2018; Mueller et al., 2018; Campbell et al., 2010). Notably, most genomic studies have emphasized classical metastatic progression; comparatively fewer have interrogated preinvasive lesions with retained epithelial histology, which are directly relevant to early dissemination (Table 1).

## Non-genetic cell-intrinsic mechanisms: epithelial extrusion and the EMT spectrum

**FIGURE 1:**
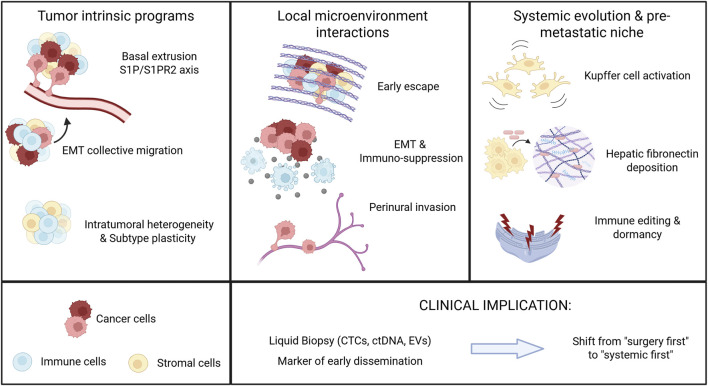
Mechanistic Landscape of Early Metastasis in Pancreatic Ductal Adenocarcinoma (PDAC). Schematic representation of the multilevel mechanisms underlying early dissemination in PDAC. Left: tumor-intrinsic programs include oncogenic *KRAS* activation driving basal extrusion through the S1P/S1PR2 axis, partial epithelial–mesenchymal transition (EMT) enabling collective migration, and subtype plasticity between classical and basal-like phenotypes that confer metastatic competence. Center: local microenvironmental interactions promote invasion through desmoplastic extracellular matrix (ECM) remodeling, interleukin-6 (IL-6)–dependent EMT and immunosuppression and perineural invasion (PNI). Right: Systemic evolution and pre-metastatic niche (PMN) formation are orchestrated by tumor-derived extracellular vesicles carrying macrophage migration inhibitory factor (MIF) that activate Kupffer cells, leading to hepatic fibronectin deposition and immune cell recruitment. Disseminated tumor cells (DTCs) persist through immune editing and endoplasmic reticulum (ER) stress–induced dormancy. Bottom: Clinical biomarkers, including circulating tumor cells (CTCs), circulating tumor DNA (ctDNA) and extracellular vesicles (EVs), may identify occult dissemination and guide a paradigm shift from a traditional “surgery-first” to a “systemic-first” management strategy in PDAC.

During homeostatic turnover, viable epithelial cells typically extrude apically into the lumen and undergo anoikis. Oncogenic KRAS perturbs this program, biasing extrusion basally so that live cells translocate into the underlying stroma, where they can survive and migrate (Slattum and Rosenblatt, 2014; Slattum et al., 2014) (Figure 1). In the pancreas, p120-catenin constrains this early invasive route through an S1P/S1PR2-dependent mechanism, indicating that epithelial integrity and lipid signaling can gate basal extrusion independently of a full EMT (Hendley et al., 2016). These data support a model in which cell-intrinsic mechanics and polarity cues enable escape from preneoplastic ducts before overt carcinoma. EMT in PDAC is not binary. Most tumors exhibit partial EMT, characterized by redistribution of junctional proteins with retention of epithelial transcripts, supporting collective invasion and circulating cell clusters; a minority display complete EMT with single-cell mesenchymal dissemination (Aiello et al., 2018). Thus, metastatic competence can emerge from finely tuned epithelial–mesenchymal plasticity rather than wholesale lineage switching.

At the transcriptomic level, genetic and epigenetic changes converge on tumor-intrinsic states that influence dissemination. Bulk and single-cell profiling delineate classical and basal-like expression programs, with frequent hybrid phenotypes and intratumoral coexistence of these states, that generates heterogeneous metabolic, epigenetic and malignant properties (Notta et al., 2016; Chan-Seng-Yue et al., 2020; Bailey et al., 2016; Moffitt et al., 2015; Cancer Genome Atlas Research Network, 2017; McDonald et al., 2017). Multi-region and single-cell studies show that classical and basal-like signatures can segregate to distinct cellular subsets within the same tumor yet remain interconvertible, implying that overall behavior reflects the dominant phenotype and its plasticity (Chan-Seng-Yue et al., 2020; Hayashi et al., 2020; Juiz et al., 2020). Proposed genomic influences include biallelic *SMAD4* loss with *GATA6* amplification (classical bias) and biallelic *TP53* and/or *CDKN2A* loss with amplified mutant *KRAS* (basal-like bias), though none of these associations is exclusive (Chan-Seng-Yue et al., 2020; N Kalimuthu et al., 2020). Importantly, subtype–metastasis relationships are context-dependent: both programs harbor features that can enhance dissemination, tumors can switch subtype during progression and mixed subtypes are common in precursor and primary lesions (Singh et al., 2024). Recent spatial-transcriptomic reconstructions further reveal microregional heterogeneity within a single metastatic organ and divergent clonal architectures across synchronous metastases, arguing against a single dominant evolutionary route from primary to metastasis and underscoring that early dissemination can arise from multiple, coexisting cell states within the same lesion (Pei et al., 2025; Steele et al., 2025).

## Microenvironmental cues enabling dissemination

### Extracellular matrix (ECM) architecture

PDAC is commonly characterized by a robust fibroinflammatory (desmoplastic) response with hyperactivated stromal fibroblasts, pronounced immunosuppression and markedly elevated ECM deposition (Stromnes et al., 2014; Neesse et al., 2015). Beyond limiting the delivery and efficacy of chemotherapy (Chauhan et al., 2013; Provenzano et al., 2012; Olive et al., 2009) and immunotherapy (Jiang et al., 2016; Feig et al., 2013), the desmoplastic ECM also contributes to the extensive metastasis frequently observed in PDAC (Lee et al., 2011; Ray et al., 2017), consistent with decreased dissemination following antifibrotic or anti-inflammatory interventions (Provenzano et al., 2012; Jiang et al., 2016). In 2022, Ray and colleagues showed that ECM organization can govern early cancer-cell escape (Ray et al., 2022). In both mouse models and human PDAC, aligned collagen fibers around pancreatic ducts—resembling tumor-associated collagen signatures (TACS) known from breast cancer—facilitated cellular extrusion and invasion even from histologically premalignant lesions. In well-differentiated PDAC, these stromal patterns enabled continued local invasion. Inducing pancreatitis in mice produced ECM architectures conducive to invasion, effectively “priming” the stroma for early dissemination, aligning with observations that pancreatitis increases early dissemination in *KRAS*-mutant models (Rhim et al., 2012). Mechanistically, pharmacologic inhibition of focal adhesion kinase (FAK) normalized stromal architecture and significantly reduced extrusion, invasion and metastasis.

### Chemokines, cytokines and ECM remodeling (including perineural invasion)

A hallmark of PDAC is its desmoplastic stroma, wherein activated pancreatic stellate cells (PSCs) and other cancer-associated fibroblasts secrete pro-metastatic factors. Key cytokines such as interleukin-6 (IL-6) and transforming growth factor-β (TGF-β) are abundant; PSC-derived IL-6 and TGF-β promote invasive progression by triggering EMT and fibrosis (Ren et al., 2018). Within the TME, IL-6 signaling activates STAT3 in cancer cells, driving EMT and recruiting immunosuppressive myeloid populations that facilitate metastasis (Wu et al., 2017). TGF-β, particularly when tumor-suppressive SMAD4 pathways are lost, induces myofibroblastic fibroblast (myCAF) expansion and an immunosuppressive niche that fosters tumor-cell escape and dissemination (Moo-Young et al., 2009; Principe et al., 2016). Chemokine gradients further guide invasion: stromal CXCL12 (SDF-1) and lymphatic endothelial CCL21 engage their cognate receptors CXCR4 and CCR7 on PDAC cells, respectively, creating chemotactic cues that direct cells into lymphatics and distant organs (Maddipati, 2025; Fink et al., 2016). In parallel, matrix metalloproteinases (MMPs) produced by tumor, stromal and inflammatory cells remodel the ECM. Tumor-associated macrophages (TAMs) secrete matrix metalloproteases (e.g., MMP-2, MMP-9) that degrade collagen and E-cadherin, enabling invasion into adjacent tissues (Xue et al., 2015).

Perineural invasion (PNI). PNI is a distinctive and prevalent feature of PDAC (70%–90% of cases) and is associated with severe neuropathic pain and poor prognosis (Maddipati, 2025; Liebl et al., 2014). PNI arises from intimate tumor–nerve interactions that co-opt developmental guidance pathways. PDAC cells frequently overexpress chemokine receptors (CX3CR1, CCR2, CXCR4, CCR5) that bind ligands presented by neurons or stromal cells (CX3CL1/fractalkine, CCL2, CXCL12, CCL5, respectively), promoting directed migration along nerve tracts (Garajova and Giovannetti, 2024). In particular, the CX3CL1–CX3CR1 axis has been implicated in neural tropism, whereby fractalkine-expressing nerves attract CX3CR1^+^ tumor cells (Marchesi et al., 2008). Beyond chemokines, aberrant axon-guidance programs contribute: semaphorins such as SEMA3D are overexpressed and promote neural invasion via plexin receptors on tumor cells (Secq et al., 2015; Jurcak et al., 2019), whereas repulsive cues such as SLIT glycoproteins and their ROBO receptors are dysregulated; loss of *SLIT2* removes a barrier to neural invasion, while restoration inhibits perineural spread (Gohrig et al., 2014; Martinelli and Real, 2019). Inflammatory cytokines further amplify these interactions: TAM-derived leukemia inhibitory factor (LIF) promotes Schwann-cell migration and neural remodeling, creating a microenvironment permissive for nerve infiltration (Albrengues et al., 2014). Spatial mapping revealed a subpopulation of transforming growth factor-β1 (TGF β1)-positive Schwann cells locate at the leading edge of NI, can be induced by TGF-β1 secreted by some TAMs and myCAFs, to promote tumor cell migration and correlate with poor survival (Chen et al., 2025). These interconnected processes are summarized in the mechanistic overview (Figure 1).

Crucially, degradation of the neural ECM and nerve-sheath structures accompanies PNI. The perineurium and endoneurium that normally protect nerves are eroded by proteases from tumor and stromal cells; TAMs upregulate MMPs around nerves, causing focal basement-membrane breakdown (Liu et al., 2022; Fu et al., 2020). Cancer-cell factors also condition the nerve microenvironment: the L1 cell adhesion molecule (L1CAM) on PDAC cells induces MMP-2 and MMP-9 via STAT3 signaling, promoting nerve infiltration; L1CAM blockade reduces PNI in mouse models (Na’ara et al., 2019). In parallel, tumor-derived TIMP1 stimulates Schwann-cell proliferation and secretion of CCL7, which in turn enhances tumor cell migration and PNI (Tian et al., 2022). Moreover, CAFs can facilitate PNI by generating a high-lactate microenvironment that induces neural invasion-associated genes, including as L1CAM and SLIT1 (Li et al., 2025). This perineural route not only aids early spread beyond the primary tumor margin but also contributes to the neural remodeling and pain that are characteristics of pancreatic cancer.

## Crosstalk and systemic evolution of the tumor

### Pre-metastatic niche priming

Mounting evidence indicates that primary tumors condition distant organs before overt spread, establishing a PMN that renders target tissues permissive to colonization (Wang et al., 2024). In PDAC, IL-6 produced within the stromal compartment stimulates hepatocytes to release serum amyloid A (SAA), driving an immunosuppressive and fibrotic state conducive to metastatic outgrowth (Lee et al., 2019). A seminal study showed that PDAC exosomes containing macrophage migration inhibitory factor (MIF) are preferentially taken up by Kupffer cells in the liver, triggering TGF-β release and fibronectin deposition by hepatic stellate cells; the remodeled matrix recruits bone-marrow–derived macrophages and creates an immunosuppressive, pro-metastatic milieu (Costa-Silva et al., 2015). Clinically, patients with Stage I PDAC who later developed liver metastases exhibited higher circulating exosomal MIF than those who did not, indicating that tumor–stroma communication can predate and enable metastatic seeding. Livers resected from patients without radiographic metastases displayed heightened inflammatory signatures with expanded myeloid and lymphoid subsets, consistent with early immune engagement at the prospective metastatic site (Bojmar et al., 2024). Within this framework, myeloid infiltration appears to be an early hallmark of the hepatic PMN, whereas T-cell infiltration later in disease may reflect countervailing antitumor activity; patients who fail to mount an intrahepatic T-cell response are more likely to progress to clinically evident liver metastasis.

### Immune editing, dormancy and reactivation of early disseminated cells

Converging mechanistic work explains how early-disseminated cells persist despite immune surveillance. Pommier et al. demonstrated that disseminated tumor cells in the liver are subject to major histocompatibility complex class I (MHC-I)–restricted T-cell killing; surviving cells are selected for an immune-evasive, quiescent state characterized by low/absent MHC-I, loss of E-cadherin and cytokeratin-19 and chronic, unresolved endoplasmic reticulum (ER) stress signaling (Pommier et al., 2018). Experimentally resolving ER stress, together with modulation of T-cell pressure, can awaken dormant DTCs to proliferate, indicating that ER-stress programs and adaptive immune editing jointly govern latency versus outgrowth. Notably, loss of E-cadherin was not accompanied by acquisition of a canonical EMT signature, supporting the view that PDAC cells do not require complete loss of epithelial identity to disseminate; partial EMT or alternative epithelial-plastic states may suffice (Hendley et al., 2016; Aiello et al., 2018).

## Discussion

Recognizing that PDAC behaves as a systemic disease from inception, conventional paradigms that prioritize upfront resection followed by adjuvant chemotherapy warrant reconsideration (Figure 1). Historical “surgery-first” strategies have produced only modest survival gains: up to 80% of patients recur after curative-intent surgery, with relapse predominantly distant rather than local (Sohal et al., 2014; Purohit et al., 2024; Groot et al., 2019; Gnerlich et al., 2012; Hishinuma et al., 2006). This pattern implies that micrometastases seeded before or at the time of surgery drive failure in most cases and explains why, despite multimodal therapy, 5-year survival after resection remains ∼20–30% (Conroy et al., 2018). Local control alone is therefore insufficient; interventions must target invisible disseminated tumor cells early. Consequently, PDAC management is shifting toward a systemic-first mindset, akin to approaches long used in breast and selected gastrointestinal cancers where (neo)adjuvant therapy is deployed to eradicate micro-metastatic disease (Bryant et al., 2025; Kim and Kin, 2021; Al-Batran et al., 2019; Cunningham et al., 2006; Conroy et al., 2021; Schmid et al., 2020).

Within this framework, neoadjuvant therapy—chemotherapy (± radiotherapy or emerging systemic agents) administered before resection—is increasingly viewed as a strategy to intercept early dissemination. The rationale is twofold. First, it may treat micro-metastases at the earliest opportunity, rather than deferring systemic therapy until after surgery. Second, it can downsize the primary tumor, increasing the probability of margin-negative resection. Consistent with this concept, Aiello et al. demonstrated in mouse models that chemotherapy even if not curative, reduced metastatic burden regardless of metastasis size (except for single disseminated cells), lowering both the frequency and size of liver metastases, and dramatically decreased circulating tumor cells (CTCs) (Aiello et al., 2016). Although initially reserved for borderline-resectable or locally advanced disease, accumulating data indicate that even clinically resectable PDAC may benefit from upfront systemic therapy. Trials and meta-analyses suggest neoadjuvant regimens are feasible and may yield outcomes comparable or superior to immediate surgery plus adjuvant therapy, with the added advantages of higher completion rates for full-dose chemotherapy and biological “selection” (progressors are spared nonbeneficial surgery) (Purohit et al., 2024; Groot et al., 2019; Al Masad et al., 2025; Henault et al., 2024; Neoptolemos et al., 2018). Early randomized evidence in borderline-resectable disease (e.g., PREOPANC) shows improved R0 rates and signals for survival benefit with neoadjuvant chemoradiation versus surgery-first (Versteijne et al., 2022). Although neoadjuvant chemotherapy with modern polychemotherapy, particularly mFOLFIRINOX, cannot yet be definitively considered superior to the surgery-first approach, pending the results of ongoing phase III trials (van Dam et al., 2023; Eade et al., 2024) (Trials.gov identifier: NCT04927780; NCT04340141), it remains a feasible, tolerable and promising option for managing resectable pancreatic adenocarcinoma (Schwarz et al., 2025). Concurrently, strategy decisions are becoming more “biology-informed”. Very high pre-treatment CA19-9 (>500 U/mL) is now widely viewed as a marker of probable occult metastasis; such patients are often steered toward neoadjuvant chemotherapy despite radiographic resectability (Isaji et al., 2018). Looking ahead, liquid biopsy biomarkers may refine this triage. CTCs integrate several hallmarks of dissemination—intravasation competence, survival in circulation and metastatic potential—and their detection in ostensibly localized PDAC correlates with early distant relapse and inferior survival in multiple cohorts (Zhang et al., 2024; Maulat et al., 2024; Franses et al., 2020). Cell-free DNA (cfDNA)—particularly its tumor-derived fraction, circulating tumor DNA (ctDNA)—offers complementary information: mutant *KRAS* ctDNA in plasma has been associated with higher recurrence risk and shorter recurrence-free survival in surgical series (Ako et al., 2021) and ctDNA kinetics may report minimal residual disease more sensitively than imaging (Bartolomucci et al., 2025). Extracellular vesicles (EVs), including exosomes, convey tumor programs that promote PMN formation; EV cargo (e.g., proteins such as glypican-1 or specific microRNAs) are under active investigation as a surrogate of aggressive biology. While none of these biomarkers is yet standard of care in PDAC, their integration could enable risk-adapted pathways, for example, systemic-first therapy or even deferral of surgery for small primaries with positive liquid biopsy, versus surgery-first for biomarker-negative cases.

Implications extend to clinical trial design. If failure is predominantly systemic and driven by early dissemination, endpoints should emphasize disease-free survival and molecular remission in addition to pathologic metrics. ctDNA (or broader cfDNA) clearance after neoadjuvant therapy or surgery is a plausible surrogate of minimal residual disease and could trigger adaptive intensification or de-escalation strategies, paralleling approaches under evaluation in colorectal cancer. Likewise, perioperative studies should incorporate longitudinal liquid biopsies (CTCs, ctDNA, EV signatures) to (i) stratify risk, (ii) assign therapy and (iii) serve as early readouts of efficacy. This philosophy is consistent with systemic-first paradigms in other solid tumors and better aligned with PDAC’s biology.

Therapeutically, targeting dissemination programs is a priority for translational research. Strategies include modulating epithelial–mesenchymal plasticity, disrupting pro-metastatic EV trafficking and enhancing immune surveillance against disseminated tumor cells. While broad “anti-metastatic” agents have not yielded benefit in PDAC, combinatorial approaches may succeed when deployed before macroscopic outgrowth, particularly in the neoadjuvant window. Given PDAC’s immunotherapy resistance, rational combinations that remodel the tumor microenvironment PMNs merit perioperative testing. Importantly, lessons from breast and colorectal cancer, where neoadjuvant and biomarker-guided designs are mature, should inform PDAC trial architecture while respecting disease-specific constraints.

In sum, PDAC’s early dissemination demands a dual shift: earlier systemic therapy to suppress micrometastases and smarter biomarkers to expose occult spread and guide timing. Neoadjuvant regimens operationalize the former; liquid biopsy (CTCs, ctDNA/cfDNA, EVs) promises the latter. Bridging basic and clinical science, by embedding mechanistic biomarkers and adaptive endpoints into perioperative trials, offers the clearest path to bending PDAC’s mortality curve, transforming a historically surgical disease into a biologically staged, systemically managed one.
